# Study of the Physicochemical and Phytochemical Parameters Together with Antibacterial Properties of Conventionally and Organically Cultivated Spirulina (*Arthrospira platensis*) in Greece

**DOI:** 10.3390/life15111761

**Published:** 2025-11-17

**Authors:** Maria-Athanasia Christodoulopoulou, Dimitrios G. Lazaridis, Maria Simoni, Eirini Intzirtzi, Vassilios K. Karabagias, Nikolaos D. Andritsos, Vassilios Triantafyllidis, Ioannis K. Karabagias

**Affiliations:** Department of Food Science and Technology, School of Agricultural Sciences, University of Patras, G. Seferi 2, 30100 Agrinio, Greece; up1076534@ac.upatras.gr (M.-A.C.); up1076474@ac.upatras.gr (D.G.L.); m.simoni@upatras.gr (M.S.); up1076528@ac.upatras.gr (E.I.); vkarampagias@upatras.gr (V.K.K.); nandritsos@upatras.gr (N.D.A.); vtrianta@upatras.gr (V.T.)

**Keywords:** *Arthrospira platensis*, spirulina, cultivation, antioxidant activity, antimicrobial activity, phenolic compounds, phycocyanin

## Abstract

Given the limited data in the recent literature regarding spirulina cultivated in Greece, the present study aimed to investigate physicochemical parameters (pH, color), antioxidant activity, and phytochemical content (total phenolic content, quercetin, caffeic acid, C-phycocyanin) of spirulina (*Arthrospira platensis*) obtained through conventional and organic cultivation methods in different forms (disk-shaped tablets, powder, and flakes) and using different solvents [water and ethanol of grape origin (EEGO)]. Furthermore, the antimicrobial activity of spirulina against selected food pathogens, namely *Salmonella* Typhimurium, *Staphylococcus aureus*, *Listeria monocytogenes*, and *Escherichia coli* O157:H7, was also investigated. Results showed that the form of spirulina and the solvent used significantly (*p* < 0.05) affected the physicochemical parameters, antioxidant activity, and phytochemical compounds. Spirulina had a neutral to alkaline pH (6.79 ± 0.02 to 8.24 ± 0.01) and a considerable number of pigments, including C-phycocyanin (0.07 ± 0.01 to 1.65 ± 0.01 mg/mL). The most pronounced effects were recorded in the case of antioxidant activity, where EEGO proved to be the best solvent for the extraction of compounds with antioxidant activity (44.37 ± 2.28% to 48.28 ± 0.39%) against the 2,2-diphenyl-1-picrylhydrazyl (DPPH) radical. In contrast, water proved to be the best solvent for the recovery of total phenolics (4.83 ± 0.55 mg/g DM to 17.07 ± 0.66 mg/g DM), primarily in spirulina powder. Regarding the antibacterial activity, spirulina, primarily in powder and tablet form (in both solvents), showed considerable inhibition zones against the studied food pathogens. The present study presents new knowledge concerning spirulina cultivated in Greece in terms of physicochemical parameters, antioxidant activity, phytochemical content, and antibacterial activity.

## 1. Introduction

*Arthrospira platensis*, or commonly, spirulina, is a microscopic, photosynthetic, filamentous, blue-green alga classified as a non-toxic cyanobacterium in the class of Cyanophyceae that belongs to the family Microcoleaceae [1]. *Arthrospira maxima* and *Arthrospira platensis*, also known as spirulina, are the two most widely studied species as they provide significant therapeutic and nutritional properties, with their difference being the thickness (i.e., *A. maxima* is thicker than *A. platensis*) [2]. *A. platensis* has a helical shape with short, multicellular trichomes with short diameter and loose coils [2]. Spirulina thrives in tropical and subtropical regions, particularly in lakes with an alkaline environment (pH = 11.0) and high concentrations of carbonates and bicarbonates. Hawaii, Mexico, Central Africa, and Asia are the regions where spirulina is mainly cultivated [1]. However, these algae can survive in extreme environments, such as the frozen lakes of Antarctica [3]. The mass cultivation and the yield of spirulina produced depend on several key factors, including the available nutrients, light, and temperature. The relatively high pH plays a crucial role in the growth of spirulina, as it inhibits the growth of other algae [4].

In recent years, it has been used in numerous applications in the industry. Spirulina has been categorized as a “superfood” given its high protein content (55–70%), along with carbohydrates, essential fatty acids, vitamins, minerals, and pigments, such as carotenoids, chlorophyll A, and phycocyanin [5]. Due to its diverse nutritional value, it has been widely used in both the food and pharmaceutical industries. In the food industry, spirulina is used as a biological food supplement, added to several foods such as bakery products [6], beverages [7], dairy products [8], supplements for athletes [9], and infant formulas [10]. On the other hand, pharmaceutical industries have developed tablets, dried powders, or capsules, which are sold as “nutritional supplements” [11]. Therefore, due to the rapid market demand and the continuous clinical trials conducted over the last decade, the U.S. Food and Drug Administration (FDA) has recognized *A. platensis* as Generally Recognized as Safe (GRAS) [12].

Numerous studies have demonstrated multiple beneficial actions, such as antioxidant, anti-inflammatory, hypolipidemic, antidiabetic, cardioprotective, and even antimicrobial effects [13,14]. Researchers have primarily focused on the natural pigment C-phycocyanin and phenolic compounds, which enhance the activity of antioxidant enzymes such as superoxide dismutase (SOD) and catalase. These enzymes contribute to the neutralization of free radicals, thereby reducing oxidative stress and protecting cells from damage [1]. It has also been documented that spirulina boosts the immune system, regulates the lipid profile (reducing total cholesterol and triglycerides), and lowers the glucose levels in humans with type II diabetes [15]. Additionally, its extracts have exhibited antimicrobial activity against foodborne pathogens such as *Escherichia coli*, *Staphylococcus aureus*, and *Listeria monocytogenes* [5]. Considering that chronic diseases remain a major global health concern, the use of natural and functional foods, such as spirulina, represents a promising complementary strategy for the prevention and management of these diseases. This approach aligns with the principle of Hippocrates: “Let food be the medicine and medicine be the food” [16].

Cyanobacteria have been widely studied for their ability to generate bioactive compounds, such as phycocyanin [4]. Phycocyanin is the main pigment of spirulina, characterized by its intense blue color, and consists of protein and non-protein components [17]. Phycocyanin may provide antioxidant activity due to its ability to scavenge oxygen and nitrogen radicals, making spirulina a healthy supplement for human health [17]. Phycocyanin is also a natural food colorant; however, its use is limited in ice cream, candies, and dairy products due to its sensitivity to heat treatment [18].

Nowadays, *A. platensis* has also been cultivated for the first time in Greece, in the region of Therma (Nigrita, Serres). While the important bioactivity and application of spirulina cultivated in different parts of the world have been previously studied, there is a need for further research on innovative applications of spirulina produced worldwide in food technology, biotechnology, environmental sustainability, and medicine, as it already emerges as a valuable natural product with wide use in nutrition and pharmacology. Herein, considering the above, we studied the physicochemical, phytochemical, and antibacterial activity profile of spirulina samples produced in different forms (tablets, powder, and flakes) through different cultivation practices (organic and conventional) using different extraction solvents (water and ethanol of grape origin). This study advances plant science or microalgal biotechnology, as this was the first time that *A. platensis* was cultivated in the region of Greece for industrial-scale production and distribution to the market. To the best of our knowledge, there is no data in the recent literature regarding the physicochemical, phytochemical, and antibacterial activity profile of spirulina cultivated in Greece, and this comprises the novelty of the present study.

## 2. Materials and Methods

### 2.1. Preparation of Spirulina Samples

**Figure 1:**
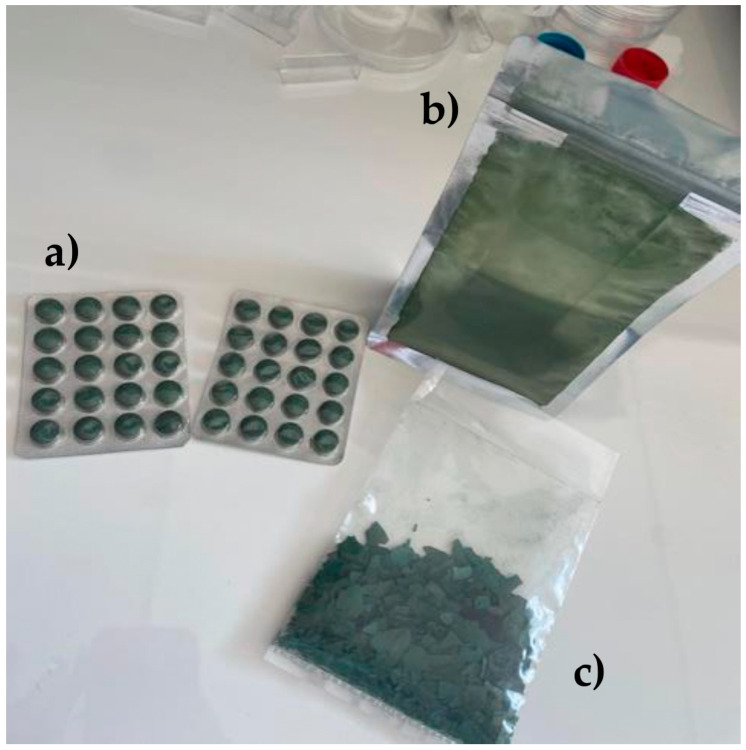
Spirulina samples in different forms: (**a**) tablet, (**b**) powder, and (**c**) flakes.

Spirulina (*A. platensis*) samples cultivated in Therma, Nigrita, Serres (Greece) (40°54′5.49″ N, 23°34′3.39″ E) were donated from a local producer in 3 different commercial forms: (1) disk-shaped tablets, (2) flakes, and (3) powder after the harvest period (March–September 2024). The most standardized form of spirulina is the tablet. For its production, the powder is compressed into a pill form through special presses. In many cases, additives (binders) are used to ensure the stability of the product, which reduces the ‘naturalness’ of the final preparation. The flakes result from drying fresh spirulina, usually naturally or thermally (low temperature or solar drier), to preserve its nutrients. Spirulina powder is also produced from dried spirulina, which is ground until it has a fine texture. Spirulina cultivated in Greece is produced exclusively in tanks with thermo-mineral waters, without chemical additives, with modern methods and constant monitoring by experts to ensure its quality. When it is ready for harvest, its biomass is pumped and separated, which is collected and spread out for natural drying. The final processing is done by the processing company Spirulina Line. The area (Therma, Nigrita, Serres), known for its rich geothermal field, offers ideal cultivation conditions, which makes this spirulina of top quality worldwide. The form of the spirulina samples is clearly represented in Figure 1. Among the samples, spirulina in flakes and powder form was from organic farming, and disk-shaped spirulina was cultivated conventionally. After the samples were collected, they were brought to the laboratory, where the flakes and disk-shaped tablets were pulverized into a fine powder using a grinding bowl. Finally, the samples were transferred to Falcon tubes and were kept in the laboratory at 25 ± 2 °C until the end of the experiments.

### 2.2. Chemicals and Reagents

All chemicals and reagents used in the present study were of analytical grade. 2,2-Diphenyl-1-picrylhydrazyl (DPPH) was purchased from TCI (Tokyo, Japan). Sodium carbonate (Na_2_CO_3_) and acetate buffer (CH_3_COONa × 3H_2_O) were purchased from Penta (Prague, Czech Republic). Folin–Ciocalteu reagent and gallic acid (3,4,5-trihydrobenzoic acid) were purchased from Sigma-Aldrich (Darmstadt, Germany). Absolute ethanol (CH_3_CH_2_OH) was purchased from Merck (Darmstadt, Germany), and caffeic acid [3-(3,4-Dihydroxyphenyl)-2-propenoic acid] 98% was purchased from BLDpharm (Kaiserslautern, Germany). Finally, quercetin [2-(3,4-dihydroxyphenyl)-3,5,7-trihydroxy-4H-chromen-4-one] (hydrate) was purchased from Cayman Chemical Company (Ann Arbor, MI, USA).

### 2.3. Extraction of Phytochemical Compounds

The phytochemical compounds of spirulina samples were extracted using two different solvents: (1) deionized water, and (2) ethanol of grape origin (EEGO) after distillation of dry white wine. EEGO is a natural and eco-friendly bi-phase solvent (aqueous-organic), occurring from the distillation of dry white wine [19]. Approximately 200 mL of commercially available dry white wine (12% *v*/*v*) was transferred to 250 mL flasks and distilled at 79 °C until 133 mL of ethanol was obtained (12 ± 0.1% *v*/*v*). The alcohol volume was measured using an alcoholmeter (GECO, Hofgeismar, Germany). It is important to mention that the EEGO solvent composition consists of water, ethanol, and acetic acid [20]. Afterward, 0.5 g of each spirulina sample was placed in a plastic Falcon tube with 50 mL of each solvent, vortexed for 5 min, and the solution was left in a dark place at 25 ± 2 °C and relative humidity of 50% for 24 h. Finally, the extracts were filtered using filter paper to obtain 1% *w*/*v* spirulina extracts, which were stored in a refrigerator (4 ± 2 °C) and used until the end of the experiments. Spirulina is a perishable product, so fresh extracts were prepared before each analysis.

### 2.4. Determination of Antioxidant Activity

#### 2.4.1. Preparation of DPPH Standard Solution

The DPPH standard solution equal to 1.01 × 10^−1^ mM was prepared by dissolving 40 mg of DPPH in 0.1 L of absolute ethanol. The volumetric flask was covered with aluminum foil and stirred in a vortex apparatus. Then, it was left in the refrigerator for 2 h to stabilize.

#### 2.4.2. Determination of In Vitro Antioxidant Activity

The antioxidant activity of spirulina extracts was measured according to Kitsios et al. [21]. Briefly, 100 μL of each extract was transferred to a plastic cuvette, followed by the addition of 1 mL of acetate buffer (100 mM) (pH 5.30 ± 0.01) and 1.9 mL of DPPH standard solution. The mixture was left in a dark place until the reaction reached a plateau (1 h), and the absorbance was measured at λ = 517 nm using a Shimadzu UV/VIS spectrophotometer (UV-1280, Shimadzu, Kyoto, Japan). The antioxidant activity was calculated using the following equation:

(1) AA% = ((A_0_ − A_t_)/A_0_) × 100

where A_0_ is the initial absorbance of the DPPH solution and A_t_ is the absorbance of DPPH after reacting with spirulina antioxidants at the plateau. Each sample was analyzed in triplicate (*n* = 3), and the results were expressed as a percentage (%) of antioxidant activity.

### 2.5. Total Phenolic Content of Spirulina Extracts

The total phenolic content (TPC) of spirulina extracts was determined according to Lazaridis et al. [22]. In a 5 mL volumetric flask, 200 μL of each spirulina extract was added, followed by the addition of 2.5 mL of deionized water and 250 μL of the Folin–Ciocalteu reagent. The mixture was left in the dark for 3 min, and then 500 μL of saturated Na_2_CO_3_ 30% *w*/*v* was added. Finally, the mixture was filled to a final volume of 5 mL with deionized water, and it was allowed to incubate in a dark place for 2 h at 25 ± 2 °C. Afterward, the absorbance of the obtained mixture was measured at λ = 760 nm. Results were expressed as mg of gallic acid equivalents per gram of dry matter (mg/g DM), after the preparation of a calibration standard curve (0–500 ppm), as follows:

(2) y = 0.004x + 0.039; *R*^2^ = 0.994

Each sample was analyzed in triplicate (*n* = 3).

### 2.6. Determination of Quercetin Content in Spirulina Extracts

The determination of the quercetin content of spirulina extracts was determined according to Lazaridis et al. [19]. In brief, 3 mL of each extract filtered through Whatman filters (0.22 μm) was transferred to a plastic cuvette, and the absorbance was measured at λ = 372 nm, after a full spectra scan in the range of 1100–190 nm. A calibration curve within the range of 0–24 mg/L was prepared by proper dilution of the standard compound solution, and the concentration was calculated using the following equation:

(3) y = 0.0709x + 0.0111; *R*^2^ = 0.9996

Each sample was analyzed in triplicate (*n* = 3), and results were expressed as mg/g DM.

### 2.7. Determination of Caffeic Acid Content

The caffeic acid content of spirulina extracts was determined using the spectrophotometric method previously introduced by Lazaridis et al. [19]. Briefly, 3 mL of each spirulina extract was filtered as mentioned above and placed in a cuvette. The absorbance was measured at λ = 325 nm, after detecting the maximum peak absorbance of caffeic acid in a full scan (1100–190 nm). A calibration curve of the standard compound was prepared (0–10 mg/L), and the concentration was calculated using the following formula:

(4) y = 0.0994x + 0.0802; *R*^2^ = 0.9976

Each sample was analyzed in triplicate (*n* = 3), and results were expressed as mg/g DM.

### 2.8. Determination of pH

For the determination of spirulina extracts, 2 mL of each extract was placed into a beaker, followed by the addition of 18 mL of deionized water, and then a portable pH meter (pHEP + HI9808, HANNA Instruments, Athens, Greece) was immersed in the solution (10% *v*/*v*). Results were expressed as pH values at 20 °C out of three replicates (*n* = 3).

### 2.9. Determination of Color Parameters

The optical parameters of spirulina extracts were determined after measuring the absorbance at λ = 420 nm (YCT: Yellow color tones), λ = 520 nm (BCT: Blue color tones), and λ = 620 nm (RCT: Red color tones) [22]. The color values were calculated using the following formulas:

(5) YCT% = (A420/CI) × 100

(6) BCT% = (A520/CI) × 100

(7) RCT% = (A620/CI) × 100

where CI: Color intensity (A420 + A520 + A620). Each sample was analyzed in triplicate (*n* = 3), and the results were expressed as percentage (%) values.

### 2.10. Determination of C-Phycocyanin Content

The C-phycocyanin content of spirulina extracts was determined using the spectrophotometric method of Bennett and Bogorad [23], according to the following equation:

(8) $$Phycocyanin\;content\;(mg/mL)=\frac{\left[{OD}_{620}-0.474\times {OD}_{652}\right]}{5.34}$$

where OD_620_ is the optical density of the sample at 620 nm and OD_652_ is the optical density of the sample at 652 nm. Each sample was filtered and analyzed in triplicate (*n* = 3), and the results were expressed as mg C-phycocyanin/mL of extract.

### 2.11. Estimation of Antibacterial Activity of Spirulina Extracts

The antibacterial activity of spirulina extracts was determined according to Koutelidakis et al. [24], with slight modifications. In brief, the antibacterial activity of each spirulina extract (ethanolic and aqueous) was tested against two Gram-negative bacteria, *Salmonella enterica* subspecies serovar Typhimurium (NCTC 12023) and *Escherichia coli* O157:H7 (NCTC 12900), and two Gram-positive bacteria, *Staphylococcus aureus* (NCTC 6571) and *Listeria monocytogenes* (ATCC-13932). *Staphylococcus aureus* and *Escherichia coli O157:H7* were purchased from Supelco^®^ Analytical Products, while *Salmonella* Typhimurium and *Listeria monocytogenes* were purchased from LGC Standards Proficiency Testing (Chamberhall Green, Bury, Lancashire, UK). Specifically, three equidistant holes (9 mm in diameter) were made on TSB (Condalab, Madrid, Spain) with Bacteriological Agar (VWR, Radnor, PA, USA) plates containing the tested strains (1% inoculum) using sterile tips. Approximately 100 μL of each extract was added to each of the three holes, respectively, and the plates were incubated at 37 °C for 24 h. The inhibition zone diameter was measured using a vernier caliper with 0.1 mm accuracy. To ensure that the antibacterial activity occurs only from the spirulina, control samples with water and EEGO were tested against all the studied bacterial strains. Results were expressed as the average ± standard deviation values of nine replicates (*n* = 9).

### 2.12. Statistical Analysis

One-way analysis of variance (ANOVA) was performed to investigate statistically significant (*p* < 0.05) differences among the average values of the determined parameters in EEGO and water in relation to spirulina type. The type of spirulina (tablet, powder, or flakes) was considered the group factor variable. In contrast, the determined parameters in EEGO and water were considered as the independent variables. Multiple comparison test (post hoc analysis) was also carried out to indicate any statistically significant (*p* < 0.05) differences between pairs of each parameter according to Tukey’s honestly significant difference (HSD). Pearson’s correlation (−1 ≤ *r* ≤ +1) bivariate statistics were carried out at the confidence level *p* < 0.05 to find positive or negative correlations among the determined parameters. Statistical analysis was done using the Statistical Package for the Social Sciences (SPSS) version 28.0 statistics software (SPSS, IBM Inc., Armonk, NY, USA, 2021).

## 3. Results

### 3.1. Antioxidant Activity

The antioxidant activity of spirulina samples is shown in Table 1. In general, EEGO achieved significantly (*p* < 0.05) better extraction of the antioxidant substances from all the samples with respect to water. Specifically, the antioxidant activity of the samples extracted with EEGO against the DPPH ranged between 44.37 ± 2.28% and 48.28 ± 0.39%, with the spirulina in flake form achieving significantly (*p* < 0.05) the highest value among the forms of tablet and powder. In contrast, the water resulted in the extraction of significantly (*p* < 0.05) higher amounts of antioxidant compounds in the powder (29.19 ± 1.37%), followed by the tablet (19.60 ± 1.23%) and the flakes (6.90 ± 0.80%).

### 3.2. Total Phenolic Content

The total phenolic content (TPC) of spirulina samples extracted with different solvents is presented in Table 1. In contrast with the previous findings, aqueous extracts showed significantly (*p* < 0.05) higher concentration in total phenolics. In brief, spirulina aqueous extract in powder form had the highest TPC (17.07 ± 0.66 mg/g DM) (*p* < 0.05), followed by the tablet form (15.40 ± 0.68 mg/g DM). Then, the spirulina powder extracted with EEGO had a TPC of 12.87 ± 0.95 mg/g DM. The aqueous extract of spirulina flakes recorded quite similar values with the tablet form extracted with EEGO (10.35 ± 0.20 mg/g DM and 10.10 ± 1.90 mg/g DM, respectively), with no statistically significant (*p* > 0.05) differences. Finally, the spirulina in flakes extracted with EEGO contained the lowest TPC value (4.83 ± 0.55 mg/g DM).

### 3.3. Secondary Metabolites

Spirulina is a source of different and major secondary metabolites, including polyphenols, sterols, phenolic acids, and flavonoids [13]. In this study, we tried to identify a phenolic acid (caffeic acid) and a flavonoid (quercetin), using the spectrophotometric method of Lazaridis et al. [19].

#### 3.3.1. Caffeic Acid

The caffeic acid content of spirulina samples is represented in Table 1. It is important to highlight that all samples had statistically significant (*p* < 0.05) differences. More specifically, the aqueous extract of spirulina in tablet form recorded the highest caffeic acid content with 2.88 ± 0.01 mg/g DM. Furthermore, spirulina in powder form had close values to the EEGO (2.69 ± 0.01 mg/g DM and aqueous (2.65 ± 0.01 mg/g DM) extracts, followed by those of the tablet form extracted with EEGO (2.34 ± 0.01 mg/g DM). Finally, the flake form of spirulina had the lowest caffeic acid content in aqueous (1.71 ± 0.01 mg/g DM) and EEGO (1.05 ± 0.01 mg/g DM) extracts.

#### 3.3.2. Quercetin

Quercetin is an abundant flavonoid with several derivatives that can be found in numerous edible plants, which possess antibacterial (see next sessions), antioxidant, and antidiabetic properties [25]. In the present study, the spirulina samples in tablet form, extracted with EEGO, recorded the highest quercetin content with 2.60 ± 0.09 mg/g DM. In the case of spirulina in powder form and in flakes, the solvent did not significantly (*p* > 0.05) affect quercetin content, and quite close values were recorded. Finally, the aqueous extract of spirulina in tablet form contained 1.99 ± 0.06 mg/g DM of quercetin.

### 3.4. C–Phycocyanin

**Figure 2:**
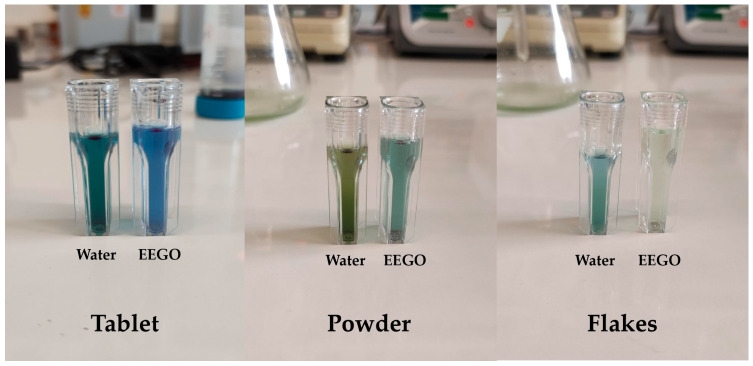
The impact of the solvent on the color of spirulina.

Phycocyanin is the most abundant pigment of *A. platensis*. The results of the C-phycocyanin content of spirulina samples extracted with different solvents are represented in Table 1. The impact of the solvent on the pigment’s extraction can also be clearly seen in Figure 2. The effective acidity of EEGO (pH = 2.16) plays an important role in the extraction of pigments, resulting in solutions with different colors [20]. The solvent did not significantly (*p* > 0.05) affect the C-phycocyanin content of spirulina in powder form (0.74 ± 0.01 mg/mL in both extracts). However, the water extract of spirulina in tablets had significantly (*p* < 0.05) higher C-phycocyanin content (1.65 ± 0.01 mg/mL) than the ethanolic extract (1.42 ± 0.01 mg/mL). In the same line of reasoning, water seemed to be a better solvent for the extraction of C-phycocyanin from spirulina in flakes, compared to EEGO (0.66 ± 0.01 mg/mL and 0.07 ± 0.01 mg/mL, respectively).

Based on Pearson’s correlation bivariate statistics, C-phycocyanin content was positively correlated with antioxidant activity (*r* = 0.786, *p* < 0.001), caffeic acid content (*r* = 0.366, *p* = 0.135), and quercetin content (*r* = 0.751, *p* < 0.001) and negatively correlated with total phenolic content (*r* = −0.017, *p* = 0.946).

### 3.5. Effective Acidity (pH)

The pH value in spirulina samples exhibited statistically significant (*p* < 0.05) differences, both within the type (tablet, powder, flakes) and between solvents (EEGO/water) (Table 2). Specifically, among the ethanol-based samples, the flakes recorded the lowest pH (6.79 ± 0.02), followed by the tablet (7.32 ± 0.02), and the highest pH values were recorded in the powder (7.77 ± 0.01). All the ethanol-extracted samples exhibited statistically significant (*p* < 0.05) differences. Furthermore, for the spirulina samples extracted with water, the flakes also had the lowest pH value (7.26 ± 0.01), followed by the tablet (8.03 ± 0.00) and the powder, which recorded the highest pH value (8.24 ± 0.01). The spirulina samples extracted with water also showed statistically significant (*p* < 0.05) differences. Overall, samples extracted with water consistently showed the highest pH compared to those with EEGO, indicating that the solvent can affect the acidity or alkalinity of the resulting extracts.

### 3.6. Yellow Color Tone

Statistical analysis showed that the yellow color tone (%YCT) of spirulina extracts recorded statistically significant (*p* < 0.05) differences between the different solvent types (EEGO/water). In samples extracted with EEGO, the tablet had the lowest YCT value (23.46 ± 0.18%), followed by flakes (38.41 ± 0.08%), while the highest YCT value was recorded in the powder (42.05 ± 0.03%). The spirulina samples extracted with EEGO showed statistically significant (*p* < 0.05) differences. In the spirulina samples extracted with water, the tablet again showed the lowest YCT value (35.80 ± 0.08%), followed by flakes (35.80 ± 0.08%), whereas the highest YCT value was recorded in powder (47.01 ± 0.08%). All samples extracted with water showed significant (*p* < 0.05) differences. The highest YCT value was recorded in the powder samples extracted with water, while the lowest was recorded in the tablet samples extracted with EEGO.

### 3.7. Blue Color Tone

The blue color tone (%BCT) of spirulina extracts demonstrated statistically significant (*p* < 0.05) differences among the different solvents (EEGO/water). In spirulina samples extracted with EEGO, the disk-shaped tablet exhibited the lowest BCT value (14.28 ± 0.17%), followed by flakes (16.04 ± 0.11%), while the highest BCT value (22.99 ± 0.09%) was recorded in powder. All samples treated with EEGO showed statistically significant (*p* < 0.05) differences. For the water-based spirulina extracts, the lowest BCT value was again recorded in the tablet (20.93 ± 0.03%), followed by flakes (24.05 ± 0.04%), and the highest BCT value was recorded in powder (24.91 ± 0.02%). It is worth noting that all the water-based extracts showed statistically significant (*p* < 0.05) differences. In conclusion, the highest BCT value was recorded in the spirulina powder extracted with water. In contrast, the lowest BCT value was recorded in spirulina samples extracted with EEGO, specifically in the tablet form.

### 3.8. Red Color Tone

The red color tone (%RCT) of spirulina extracts also varied significantly (*p* < 0.05) among the different solvents (EEGO/water). For the spirulina samples extracted with EEGO, the lowest RCT value was recorded in powder (34.94 ± 0.08%), followed in increasing order by the flakes (45.54 ± 0.03%), whereas the highest value was recorded in the tablet (62.25 ± 0.35%). In addition, in spirulina samples extracted with water, the lowest RCT value was recorded in powder (27.99 ± 0.18%), followed by the flakes (33.76 ± 0.05%), whereas the highest value was recorded in the tablet (43.28 ± 0.06%). Overall, the highest RCT was recorded in spirulina tablets extracted with EEGO, and the lowest was recorded in the powder extracted with water.

### 3.9. Estimation of Antibacterial Activity

**Figure 3:**
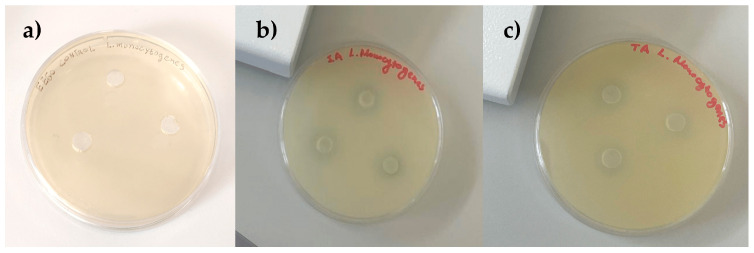
Representative inhibition zones of spirulina extracted with ethanol of grape origin (EEGO) against *Listeria monocytogenes*: (**a**) control, (**b**) powder form, and (**c**) tablet form.

The antibacterial activity of spirulina samples is represented in Table 3 and Figure 3. As we mentioned earlier, spirulina contains polyphenols, flavonoids, and antioxidant substances that generally exhibit antimicrobial activity and limit the growth of microorganisms. Considering the inhibition zones of spirulina extracted with water and EEGO, against Gram-negative (−) bacteria, these ranged between 0.00 and 2.30 ± 0.07 mm against *Salmonella* Typhimurium, and 0.14 ± 0.02–1.86 ± 0.14 mm against *E. coli* O157:H7. Similarly, their inhibitory effect against Gram-positive (+) strains ranged between 0.00–3.18 ± 0.17 mm against *Staphylococcus aureus*, and 0.17 ± 0.01–4.22 ± 0.10 mm against *Listeria monocytogenes*.

## 4. Discussion

It is widely known that plant foods, including algae, are a rich source of antioxidant compounds [26], and spirulina is a well-studied natural food supplement with high antioxidant activity [14,26]. In this regard, the solvent and its characteristics (i.e., polarity) can easily influence the extraction of phytochemical compounds, of which solubility can be affected [26,27]. Specifically, in our case, the use of a bi-polar solvent (EEGO) resulted in better extraction of bioactive compounds that possess antioxidant activity. For instance, a recent work that studied spirulina samples from different regions in various forms reported that ethanolic extracts of spirulina in tablet form achieved 46.59% radical scavenging against DPPH, while that of the powder form was 35.61%. Moreover, the geographical location where spirulina was cultivated significantly affected its antioxidant power, among the different regions (USA, China, and Europe) [28]. These results agree with the findings of the present study. Furthermore, Machu et al. [14] reported that the aqueous extracts of Indian spirulina samples had a considerable antioxidant activity (0.76 μmol of ascorbic acid/g). Furthermore, Moroccan spirulina samples extracted with methanol, acetone, and ethanol inhibited the DPPH by 40.44 ± 2.66%, 39.77 ± 0.69%, and 35.41 ± 0.45%, respectively [29]. In addition, microcapsules that were developed with Arabic gum and whey protein isolate exhibited 56.38 ± 0.19% antioxidant activity against DPPH [30]. Overall, the results in the literature confirm the presence of a significant number of antioxidants in *A. platensis*, which is consistent with the present work.

Spirulina contains high amounts of phycocyanin, which is its main antioxidant with health benefits [26]. In this context, we can clearly understand that the solvent used and the type of sample, along with the physiological and nutritional variations, can influence the quantity of phenolic compounds. A previous study in the literature reported that aqueous extracts of spirulina contained higher concentrations of phycocyanin, carbohydrates, proteins, and free amino acids than spirulina ethanolic extracts [31], given their ability to react with the Folin–Ciocalteu reagent [32]. Moreover, phycocyanin is insoluble in ethanol [33]. Machu et al. [14] recorded total phenolic content values in A. platensis that ranged between 43.2 ± 1.0–17.0 ± 0.5 mg GAE/g, in relation to the used solvent and the extraction method. Alshuniaber et al. [34] obtained similar values in total phenolic content that ranged between 7.80 ± 0.15–44.48 ± 1.71 mg GAE/g. Likewise, a similar work studied A. platensis samples from Morocco, extracted with methanol, acetone, and ethanol, and reported values of 7.00 ± 0.07 mg GAE/g, 4.65 ± 0.27 mg GAE/g, and 3.72 ± 0.22 mg GAE/g, respectively [29].

Caffeic acid is a hydroxycinnamic acid that naturally occurs in almost all plants, well-known for its antioxidant and anti-inflammatory activity [35], and is generally associated with antibacterial properties [36]. However, since *A. platensis* is a bacterium and not a plant, its presence occurs after biosynthesis through the shikimic acid pathway, where phenylalanine is deaminated to cinnamic acid, before being transformed to caffeic acid [37]. For example, Kuntzler et al. [38] previously identified caffeic acid in *A. platensis* at a respected concentration (0.347 mg/g), using high-performance liquid chromatography (HPLC). In the same line of reasoning, Pyne and Paria [39] determined caffeic acid in spirulina samples after supercritical-CO_2_ extraction, with the concentrations ranging between 12.17 ± 4.78–72.11 ± 1.05 μg/g, depending on the extraction parameters used (i.e., pressure, extraction time, temperature). Another interesting research was that of El-Baky et al. [40], where researchers tried to illustrate the phenolic synthesis in Spirulina maxima during the growth stage, cultivating it in four different NaNO_3_ concentrations. The caffeic acid content of the samples ranged between 1.80 and 7.90 mg/g (similar to the findings of the present study), while it seems from these results that the phenolic composition of algae products can be easily affected by the cultivation practice.

Regarding quercetin content, the present results conform to previous studies concerning spirulina, where researchers recorded quercetin content values ranging between 1.10 and 6.30 mg/g [40,41]. Quercetin was also identified in *A. platensis* samples from Morocco [42] and Turkey [43], in a concentration of 0.26 mg/g.

The results of C-phycocyanin content in spirulina are in agreement with previous studies. Su et al. [44] reported C-phycocyanin values that ranged between 0.3 and 2.0 mg/mL depending on the time of extraction and pH of the solvent. Moreover, Aftari et al. [45] obtained similar values (1.4–2.2 mg/mL) when they ultrasonically extracted C-phycocyanin in different extraction times (1–10 min). The pH of the solvent plays the most important role in its extraction. The best pH conditions for the extraction of C-phycocyanin were reported to be at pH = 6–7, while C-phycocyanin is unstable at pH above 8 and below 5 (EEGO pH = 2.16, deionized water pH = 7) [44,46]. Furthermore, the extraction temperature is crucial, as in temperatures above 50 °C, the denaturation of C-phycocyanin proteins’ structure occurs, leading to changes in their color and chromophore stability [46,47].

In terms of physicochemical parameters, other studies concerning spirulina samples extracted with deionized water reported pH values ranging between 7.1 ± 0.0 in powder and ranging between 7.2 ± 0.1 in tablets [48]. However, spirulina needs an alkaline environment with a pH ranging from 8.3 to 11 to grow [49]. In our opinion, the alkaline pH of the extracts is strongly linked with the growth environment of *A. platensis*. High pH environment favors the production of bicarbonate/carbonate substances, which *A. platensis* utilizes, due to the formation of CO_2_/H_2_CO_3_/HCO^3−^/CO_3_^−2^ [50].

Based on a previous study conducted on eight different samples of spirulina powder and flakes extracted with acetone [51], it was reported that the powder was richer in carotenoids (yellow color tone) compared to the flakes. These findings are consistent with the present results obtained from spirulina extracts using EEGO and water. Color is relevant to the consumer’s acceptability, along with pigments that play an important role in the overall phytochemical and physicochemical composition of various foods.

Regarding the pigment profile, in a previous study, the phytocyanin content of spirulina was evaluated using different solvents, including distilled water, sodium acetate buffer, and phosphate buffer. The experiments were conducted on various forms of spirulina biomass, including dried and wet samples. The results demonstrated that extraction with distilled water produced the highest blue color tone, which is in agreement with our findings showing that water extraction yields higher blue tone intensity compared to ethanol [52]. Additionally, according to another study, the phycocyanin content in spirulina may be reduced using a heat pump, as is done for powder production, which indicates that both the powder and the flakes have a lower blue color tone when extracted in water [53].

Phycoerythrin, one of the main phycobiliproteins found in spirulina, is responsible for its characteristic red pigment. In the present study, the ethanolic extract of the tablets exhibited a high red color tone, which, according to the literature, can be attributed to ethanol’s enhanced ability to extract phycobiliproteins such as phycoerythrin [54].

Finally, in most cases, the solvent did not significantly (*p* > 0.05) affect the antibacterial activity of Greek spirulina. However, the highest inhibition zones were recorded for the Gram-positive bacteria. Microalgae are a rich source of fatty acids, especially polyunsaturated fatty acids (PUFAs), and they are one of the most abundant and underappreciated antimicrobial compounds [36,55]. PUFAs have higher antibacterial activity compared with saturated fatty acids, which is mainly attributed to their double bonds [56]. It should be noted that spirulina is rich in γ-linoleic acid, an antibacterial fatty acid, with a concentration reaching 36% of the total PUFAs. Gamma-linolenic acid has high antibacterial activity only against Gram-positive bacteria, and not against Gram-negative bacteria [55]. Abdel-Moneim et al. [5] extracted the bioactive compounds from spirulina using organic solvents (methanol, acetone, and hexane) in different concentrations. The authors tested the extracts against the same bacteria we investigated in our study, recording remarkable inhibition zones that ranged between 10.6 and 28.0 mm. Furthermore, the antibacterial activity of *A. platensis* was also reported by Alshuniaber et al. [34]. The antimicrobial activity of *A. platensis* can also be attributed to phycocyanin, which is another major antibacterial and antifungal molecule [36]. In this context, we should not forget that in the present study, spirulina from organic and conventional cultivation was investigated. Therefore, we postulate that the differences in the aqueous extracts of these samples may also be attributed to the different cultivation practices and post-harvest processing (tablet, flakes, and powder). Considering the previous studies in the literature, we should stress that the present study contributes further to the field of spirulina research as it investigates the physicochemical, phytochemical, and antibacterial activity profile of spirulina cultivated in Greece and produced in different forms through the analysis of their extracts using green solvents (water and ethanol of grape origin). The data derived from the present study could probably set the basis for new research or innovative applications of spirulina formulations (i.e., bioactive supplements) in the food or pharmaceutical sectors, with priority given to the beneficial recovery of specific phytochemicals in extract forms and further usage in foods or medicinal products under proper processing.

## 5. Conclusions

The physicochemical parameters, such as pH and color, antioxidant activity, and phytochemical content of spirulina (*A. platensis)* obtained through conventional and organic cultivation in different forms (disk-shaped tablets, powder, and flakes) proved to be characteristic. More precisely, these parameters were significantly affected by the extraction solvent (water or EEGO). In addition, spirulina samples had considerable antimicrobial activity against *Salmonella* Typhimurium, *Staphylococcus aureus*, *Listeria monocytogenes*, and *Escherichia coli* O157:H7. This study presents new knowledge regarding the antioxidant and antimicrobial activities of spirulina cultivated in Greece, as it is the first work in the literature that reports on these experimental data concerning the specific spirulina samples studied. In this context, the study may be a useful guide for practitioners or farmers to flourish the cultivation of spirulina in different parts of Greece, and especially expand the organic cultivation of spirulina, which showed a considerable antioxidant activity and total phenolic content, so the product can be more attractive in the global market. In addition, the data originating from this research may set a practical basis for the preparation of spirulina formulations in food or medicinal products, with emphasis placed on the development of innovative products with spirulina bioactive extracts. Apart from the contribution of the present study to the field of spirulina research, there is a need for the complete characterization and determination of more bioactive phytochemicals found in spirulina using more robust instrumental methods of analysis, such as high-pressure liquid chromatography coupled with mass spectrometry (LC/MS–MS).

## Figures and Tables

**Table 1:** Antioxidant activity and phytochemical content of spirulina samples extracted with different solvents.

Samples	Solvent	Antioxidant Activity (%)	Total Phenolic Content (mg/g DM)	Caffeic Acid Content (mg/g DM)	Quercetin Content (mg/g DM)	C-Phycocyanin Content (mg/mL)
Τablets	EEGO	44.37 ±2.28 ^a^	10.10 ± 1.90 ^a^	2.34 ± 0.01 ^a^	2.60 ± 0.09 ^a^	1.42 ± 0.01 ^a^
Powder	EEGO	47.71 ± 1.28 ^a^	12.87 ± 0.95 ^b^	2.69 ± 0.01 ^b^	2.30 ± 0.03 ^b^	0.74 ± 0.01 ^b^
Flakes	EEGO	48.28 ± 0.39 ^ab^	4.83 ± 0.55 ^c^	1.05 ± 0.01 ^c^	1.40 ± 0.02 ^c^	0.07 ± 0.01 ^c^
Τablets	Water	19.60 ± 1.23 ^c^	15.40 ± 0.68 ^d^	2.88 ± 0.01 ^d^	1.99 ± 0.06 ^d^	1.65 ± 0.01 ^d^
Powder	Water	29.19 ± 1.37 ^d^	17.07 ± 0.66 ^d^	2.65 ± 0.01 ^e^	2.40 ± 0.01 ^b^	0.74 ± 0.01 ^b^
Flakes	Water	6.90 ± 0.80 ^e^	10.35 ± 0.20 ^a^	1.71 ± 0.01 ^f^	1.41 ± 0.02 ^c^	0.66 ± 0.01 ^e^

EEGO: Ethanol of grape origin. DM: dry mass. Different letters in each column indicate statistically significant (*p* < 0.05) differences according to Tukey’s honestly significant difference (HSD) test.

**Table 2:** Effective acidity (pH) and color of spirulina samples extracted with different solvents.

Samples	Solvent	pH	YCT (%)	BCT (%)	RCT (%)
Tablets	EEGO	7.32 ± 0.02 ^a^	23.46 ± 0.18 ^a^	14.28 ± 0.17 ^a^	62.25 ± 0.35 ^a^
Powder	EEGO	7.77 ± 0.01 ^b^	42.05 ± 0.03 ^b^	22.99 ± 0.09 ^b^	34.94 ± 0.08 ^b^
Flakes	EEGO	6.79 ± 0.02 ^c^	38.41 ± 0.08 ^c^	16.04 ± 0.11 ^c^	45.54 ± 0.03 ^c^
Tablets	Water	8.03 ± 0.00 ^d^	35.80 ± 0.08 ^d^	20.93 ± 0.03 ^d^	43.28 ± 0.06 ^d^
Powder	Water	8.24 ± 0.01 ^e^	47.01 ± 0.08 ^e^	24.91 ± 0.02 ^e^	27.99 ± 0.18 ^e^
Flakes	Water	7.26 ± 0.01 ^f^	42.19 ± 0.02 ^f^	24.05 ± 0.04 ^f^	33.76 ± 0.05 ^f^

YCT: Yellow color tone, BCT: Blue color tone, RCT: Red color tone. Different letters in each column indicate statistically significant (*p* < 0.05) differences according to Tukey’s honestly significant difference (HSD) test.

**Table 3:** Antibacterial activity of spirulina samples extracted with different solvents.

		Inhibition Zone (mm)			
Samples	Solvent	*Salmonella* Typhimurium	*Staphylococcus aureus*	*Listeria monocytogenes*	*E. coli* O157:H7
Tablets	EEGO	0.00 ± 0.00 ^a^	2.46 ± 0.06 ^a^	1.65 ± 0.14 ^a^	1.54 ± 0.09 ^a^
Powder	EEGO	2.15 ± 0.05 ^b^	2.55 ± 0.04 ^a^	2.88 ± 0.56 ^b^	1.86 ± 0.14 ^b^
Flakes	EEGO	0.51 ± 0.11 ^c^	0.00 ± 0.00 ^b^	0.23 ± 0.05 ^c^	0.32 ± 0.10 ^c^
Tablets	Water	2.30 ± 0.07 ^b^	3.18 ± 0.17 ^c^	4.22 ± 0.10 ^d^	0.32 ± 0.02 ^c^
Powder	Water	0.24 ± 0.04 ^d^	0.41 ± 0.01 ^d^	0.17 ± 0.01 ^c^	0.14 ± 0.02 ^c^
Flakes	Water	0.00 ± 0.00 ^a^	1.81 ± 0.03 ^e^	1.30 ± 0.04 ^a^	0.22 ± 0.03 ^c^

EEGO: Ethanol of grape origin. Different letters in each column indicate statistically significant (*p* < 0.05) differences according to Tukey’s honestly significant difference (HSD) test.

## Data Availability

The original contributions presented in this study are included in the article. Further inquiries can be directed to the corresponding author.
